# Advanced Regenerative Drainage and Immunomodulatory Protocol for Dermal Filler–Induced Skin Necrosis: A Prospective Clinical Case Series

**DOI:** 10.1111/jocd.70847

**Published:** 2026-04-07

**Authors:** Macarena Olivares, Diego Araya, Eloisa Forero, María José Benoit, Victor Mercado

**Affiliations:** ^1^ Aesthetic Department Instituto Chileno de Rejuvenecimiento y Optimización de Medicina Estética Santiago Chile; ^2^ Aesthetic Department Faculdade do Oeste Paulista Facop Bauru Brazil; ^3^ Aesthetic Department Advanced Aesthetic Academy Santiago Chile; ^4^ Otolaryngology, Instituto de Neurorrehabilitación y Equilibrio Viña del Mar Chile

**Keywords:** ARDIP, hyaluronic acid, hyaluronidase, multimodal treatment, tissue necrosis, vascular complication

## Abstract

**Background:**

The widespread use of hyaluronic acid (HA) dermal fillers has been accompanied by an increase in vascular complications, including ischemia and cutaneous necrosis. Although uncommon, filler‐induced vascular compromise represents one of the most severe adverse events in aesthetic medicine and requires immediate recognition and intervention. Conventional management strategies based primarily on hyaluronidase may be insufficient in advanced ischemic stages, where inflammatory amplification and microvascular dysfunction contribute to progressive tissue injury.

**Objective:**

To evaluate the clinical outcomes of the Advanced Regenerative and Immunomodulatory Drainage Protocol (ARDIP), a multimodal therapeutic approach designed to restore tissue perfusion, reduce inflammatory burden, and promote regenerative healing in patients with facial skin necrosis secondary to hyaluronic acid dermal fillers.

**Methods:**

A prospective, non‐randomized interventional case series was conducted including 20 patients presenting with vascular occlusion (VO) and cutaneous necrosis following hyaluronic acid injections. Lesions were classified according to a staged ischemic progression system (VO Stages III–Vb). All patients were treated using the ARDIP, which integrates high‐dose hyaluronidase reperfusion, topical fibrinolytic therapy, controlled mechanical drainage through micropuncture techniques, and stage‐adjusted systemic and regenerative adjuvant therapies. Clinical outcomes were assessed through standardized photographic documentation, healing time, and the Global Aesthetic Improvement Scale (GAIS).

**Results:**

All patients completed treatment and follow‐up. Complete tissue recovery without visible scarring was achieved in 95% of cases. Mean healing time was 15 ± 2.6 days, with faster recovery in early stages. The mean GAIS score was 1.05 ± 0.22, indicating exceptional aesthetic improvement. No systemic or local adverse events were observed.

**Conclusions:**

ARDIP appears to be a safe and effective multimodal strategy for the management of filler‐induced skin necrosis, addressing both mechanical vascular obstruction and the associated inflammatory cascade.

## 1 Introduction

Dermal filler injections have become one of the most commonly performed procedures in aesthetic medicine due to their effectiveness in facial rejuvenation and their minimally invasive nature. Hyaluronic acid (HA)‐based fillers are widely used because of their favorable safety profile and reversibility with hyaluronidase (Hyal). However, despite their overall safety, serious complications may occur, including vascular occlusion resulting from accidental intravascular injection or external vascular compression. When blood flow is compromised, tissue ischemia may rapidly develop and can progress to cutaneous necrosis if not promptly recognized and treated [1, 2, 3].

Although the reported incidence of these complications is low, it is not negligible and is likely underestimated. Observational studies and systematic reviews estimate that the incidence of cutaneous ischemic events ranges from 1 in 2000 to 1 in 10 000 procedures. Technique‐related differences have been reported, with a significantly lower incidence when cannulas are used compared to needles. Additionally, a 30‐fold increase in filler‐induced cutaneous ischemic injuries has been documented between 2000 and 2020, with hyaluronic acid fillers being the most frequently implicated [3, 4, 5].

From a pathophysiological perspective, interruption of blood flow leads to acute tissue hypoxia, forcing cells to shift from mitochondrial oxidative phosphorylation to anaerobic metabolic pathways, primarily glycolysis, for ATP production. This compensatory mechanism is transient and energetically inefficient, resulting in rapid ATP depletion and lactate accumulation, leading to intracellular acidosis. When ischemia persists, these changes become irreversible, culminating in cell death by necrosis, typically coagulative necrosis in cutaneous tissue [6].

In the context of dermal fillers, this clinicopathological process falls within the spectrum of embolia cutis medicamentosa, also known as Nicolau syndrome, characterized by five stages of vascular occlusion: acute ischemia or pallor, livedo reticularis, pustule formation, coagulation, desquamation or early eschar formation, and ultimately cutaneous necrosis [2, 3].

We conducted a prospective, non‐randomized interventional case series to evaluate the clinical outcomes of the ARDIP in patients with HA filler–induced cutaneous necrosis.

## 2 Materials and Methods

### 2.1 Study Population and Design

Inclusion criteria included clinical diagnosis of vascular compromise with cutaneous ischemia or necrosis following hyaluronic acid injection and absence of ophthalmologic involvement.

A prospective, non‐randomized interventional study including 20 (17 females, 3 males) consecutive patients presenting with facial skin necrosis secondary to hyaluronic acid dermal filler injection with lesions predominantly located in high‐risk vascular territories including glabella, nasal dorsum, nasal tip, alar region, nasolabial fold, upper and lower lip, midface, and temporal fossa (Table 1). Onset ranging from early ischemic signs to established necrosis presented in Table 2 (VO Stage I‐V according to internal clinical) [2].

**TABLE 1:** Patient characteristics and lesion VO stage.

Number patient	Gender	Area	VO stage
Patient 1	Female	Glabella	Stage III
Patient 2	Female	Glabella	Stage III
Patient 3	Female	Mid nasal dorsum, tip and upper lip	Stage III
Patient 4	Female	Nasal dorsum and left genian area	Stage III
Patient 5	Female	Glabella, nasal dorsum and left ala	Stage IV
Patient 6	Female	Right midface	Stage IV
Patient 7	Male	Glabella	Stage IV
Patient 8	Female	Glabella	Stage IV
Patient 9	Female	Nasal dorsum, tip, base and upper lip	Stage IV
Patient 10	Female	Glabella, nasal dorsum, upper lip	Stage Va
Patient 11	Male	Nasal dorsum, tip and base	Stage Va
Patient 12	Female	Right nasal dorsum and tip	Stage Va
Patient 13	Male	Upper right nasal dorsum	Stage Va
Patient 14	Female	Nasal dorsum, tip, base and upper lip	Stage Va
Patient 15	Male	Glabella	Stage Va
Patient 16	Female	Nasal dorsum, tip and base	Stage Vb
Patient 17	Female	Left lower lip	Stage Vb
Patient 18	Female	Left ala and left nasolabial fold	Stage Vb
Patient 19	Female	Nasal tip	Stage Vb
Patient 20	Female	Temporal fossa	Stage Vb

**TABLE 2:** Distribution of vascular occlusion stages.

Variable	*n*
VO stages	
Stage III	4
Stage IV	5
Stage Va	6
Stage Vb	5
Total patients:	20

*Note:* Distribution of patients according to VO stages prior to initiation of the ARDIP. VO stages were classified as Stage III (*n* = 4), Stage IV (*n* = 5), Stage Va (*n* = 6), and Stage Vb (*n* = 5).

Our study excluded patients with decompensated chronic diseases, systemic infections, autoimmune disorders, coagulopathies, pregnancy, and known allergies to hyaluronidase or topical heparin (Table 3).

**TABLE 3:** Exclusion criteria.

Decompensated chronic disease
Systemic infection
Autoimmune disorders
Coagulopathies
Pregnancy
Known allergy to hyaluronidase or topical heparinoid

### 2.2 Management ARDIP

All patients were treated according to the ARDIP, a multimodal therapeutic strategy designed to restore tissue perfusion, modulate inflammation, and promote regenerative tissue repair (Table 4).

**TABLE 4|:** ARDIP.

Step	Intervention	Clinical objective
Step 1. Thrombus modulation	Application of a topical fibrinolytic gel containing mucopolysaccharide polysulfate or heparinoid derivatives.	To soften thrombotic material, improve microcirculatory permeability, and facilitate subsequent drainage.
Step 2. Enzymatic decompression	Hyaluronidase microinjections administered with a 30‐gauge needle (0.05 mL per injection point).	To promote enzymatic degradation of intravascular or extravascular hyaluronic acid deposits and reduce mechanical vascular obstruction.
Step 3. Manual drainage	Manual compression and drainage of the affected area.	To evacuate necrotic debris and sero‐hematic fluid while promoting restoration of capillary perfusion.
Step 4. Regenerative therapies	Adjunctive regenerative approaches including hyperbaric oxygen therapy, ozone therapy, photobiomodulation, polynucleotides, and exosomes.	To stimulate cellular activity, enhance angiogenesis, support extracellular matrix restoration, and modulate the inflammatory response.

*Note:* Used for the management of ischemic and necrotic complications secondary to dermal fillers. The protocol integrates thrombus modulation, enzymatic decompression, manual drainage, and regenerative therapies aimed at restoring microcirculation and promoting tissue regeneration.

The ARDIP is specifically indicated for advanced vascular complications (VO stages III–Vb), typically beyond 48 h after onset, when tissue injury has progressed beyond the reversible ischemic phase and hyaluronidase alone is insufficient.

Clinically, these stages are characterized by persistent ischemia, early necrotic changes, and progression from pustular lesions to necrotic eschar formation. In this context, a multimodal strategy integrating drainage, immunomodulation, and regenerative therapies is required to restore tissue viability and limit necrosis progression.

The time interval between filler injection and treatment initiation ranged from early intervention within hours of symptom onset to delayed presentations beyond 48–72 h. In early stages (within 24–48 h), patients were managed according to established protocols for acute vascular occlusion, including repeated high‐dose hyaluronidase administration, massage, local heat application, and supportive reperfusion measures.

**FIGURE 1:**
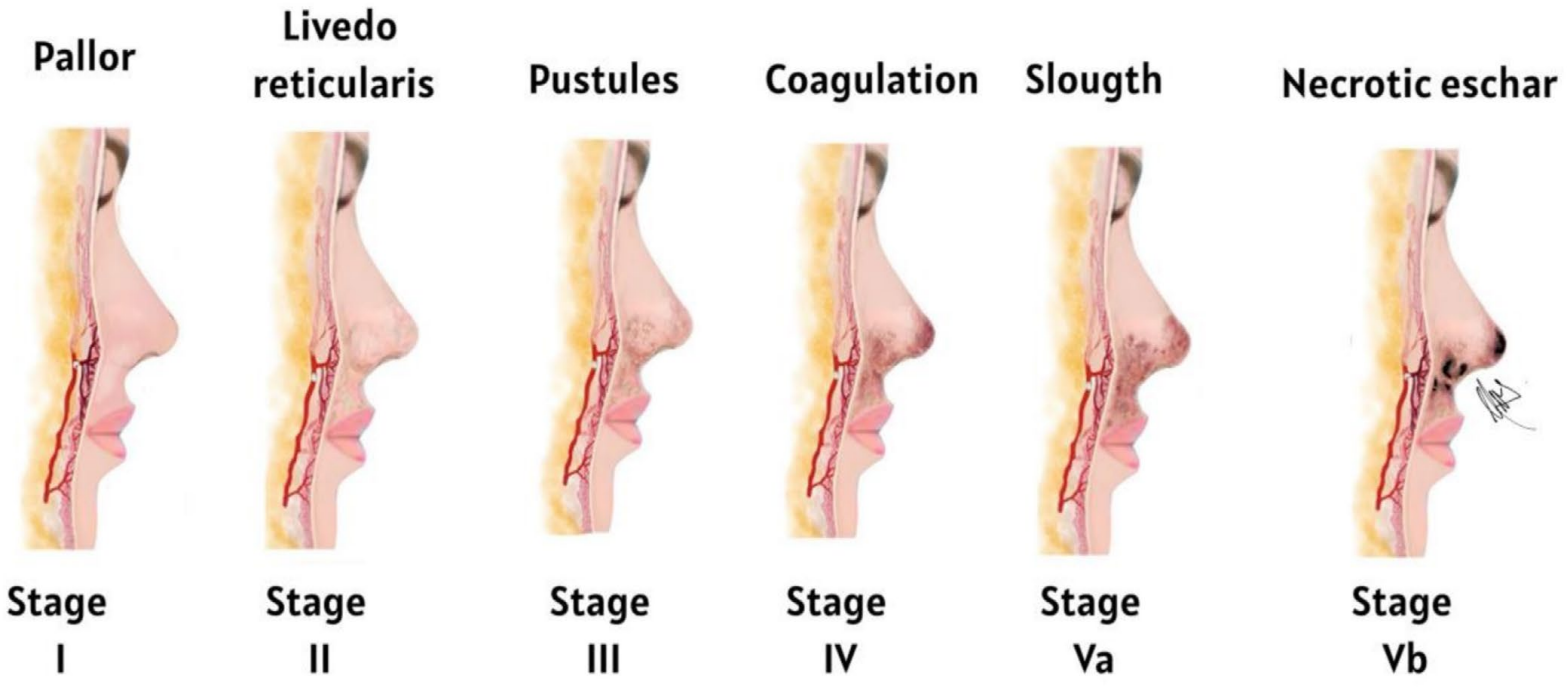
Clinical progression of vascular compromise following arterial obstruction induced by hyaluronic acid dermal fillers. The schematic diagram illustrates the sequential stages of ischemic skin injury, ranging from early vascular compromise (pallor and livedo reticularis) to progressive tissue damage characterized by pustule formation, coagulation dysfunction, slough development, and eventual necrotic eschar formation (VO Stages I–Vb). Illustration created by author.

Vascular occlusion stages were classified as follows: early stages (I–II) presented with pallor or livedo reticularis and delayed capillary refill; stage III was associated with pustular changes; stage IV with progressive ischemia; and advanced stages (Va–Vb) with early slough formation and established necrotic eschar (Figure 1).

The ARDIP followed a stepwise approach including topical fibrinolytic agents and high‐dose hyaluronidase microinjections (1500 IU per session; Liporase, Daehan New Pharm, South Korea). Hyaluronidase was administered in repeated doses every 20–30 min based on capillary refill and clinical response until reperfusion was achieved, with additional doses administered as clinically indicated.

Manual drainage was performed using gentle, controlled, atraumatic low‐pressure techniques adapted to fragile ischemic tissue. The aim was to reduce interstitial pressure, facilitate venous outflow, and improve microvascular perfusion.

Adjuvant systemic therapies were administered according to clinical severity and included short‐course oral corticosteroids (prednisone 40 mg daily for 5 days), sildenafil (50 mg daily for 2 days), and low‐dose aspirin (75 mg daily up to 300 mg).

In selected advanced cases, additional supportive therapies such as hyperbaric oxygen therapy (3 ATA, 7 sessions), photobiomodulation, and ozone therapy were incorporated.

Regenerative support was introduced in advanced or reparative stages. Initial treatment included enzymatic debridement with collagenase (0.5 mL) in VO stages Va–Vb, followed by ozone therapy (35–40 μg/mL), perilesional polydeoxyribonucleotide (PDRN) injections (20 mg/mL; 0.2 mL), and infrared photobiomodulation (808 nm, 4 J/cm^2^).

**FIGURE 2:**
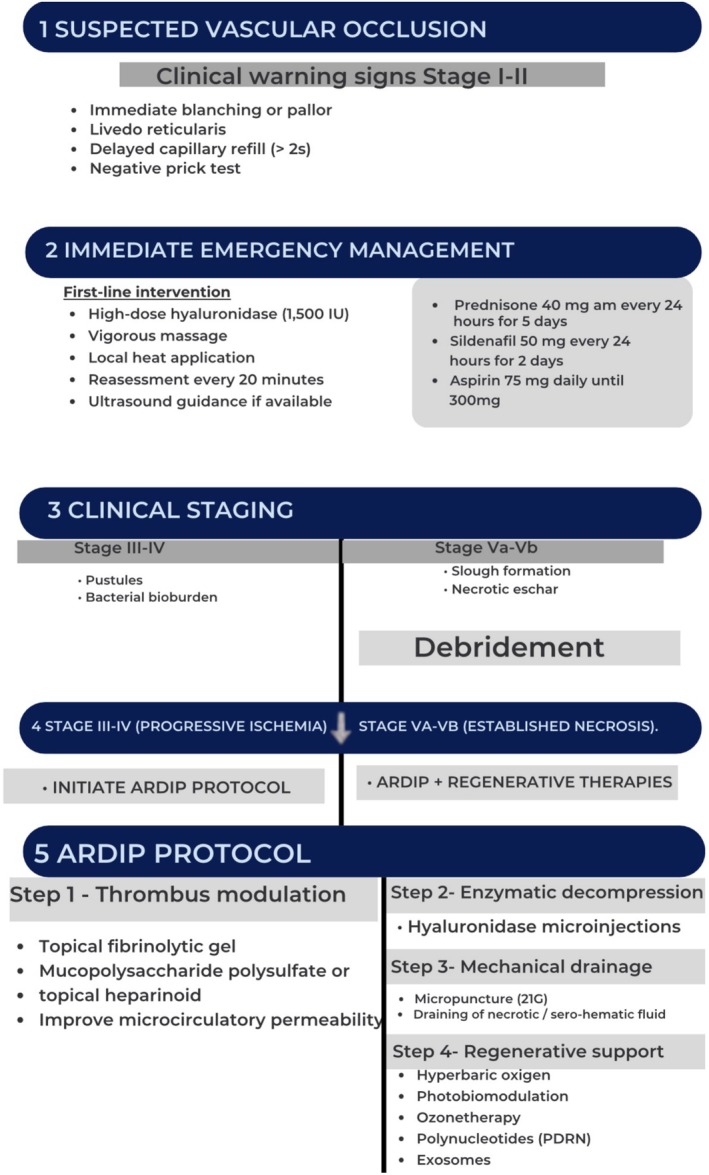
Clinical staging and management algorithm for hyaluronic acid–induced vascular occlusion. The schematic diagram illustrates the staged progression of vascular compromise (Stages I–Vb) and the corresponding therapeutic approach, including early hyaluronidase reperfusion and implementation of the ARDIP with adjunctive therapies in advanced stages. Diagram created by author.

Subsequent sessions were performed at 10‐day intervals using a multimodal regenerative approach including ozone therapy (45 μg/mL), PDRN, photobiomodulation, and *Panax ginseng* –derived exosomes administered via microneedling (Figure 2).

### 2.3 Adverse Event Reporting

No systemic or local adverse events were observed during the therapeutic process. Patients receiving systemic corticosteroids combined with aspirin were prescribed prophylactic gastric protection.

### 2.4 Photo Documentation

High‐resolution digital photographs were obtained using an iPhone 16 Pro Max camera. All patients underwent standardized clinical evaluation and photographic documentation at baseline and during follow‐up.

### 2.5 Measure Resolution

The Five‐point Global Aesthetic Improvement Scale (GAIS) was applied at treatment completion. Scores ranged from 1 (exceptional improvement) to 5 (worsened condition) [6].

### 2.6 Histopathological Evaluation

Biopsy was not included as part of the standard ARDIP management.

**FIGURE 3:**
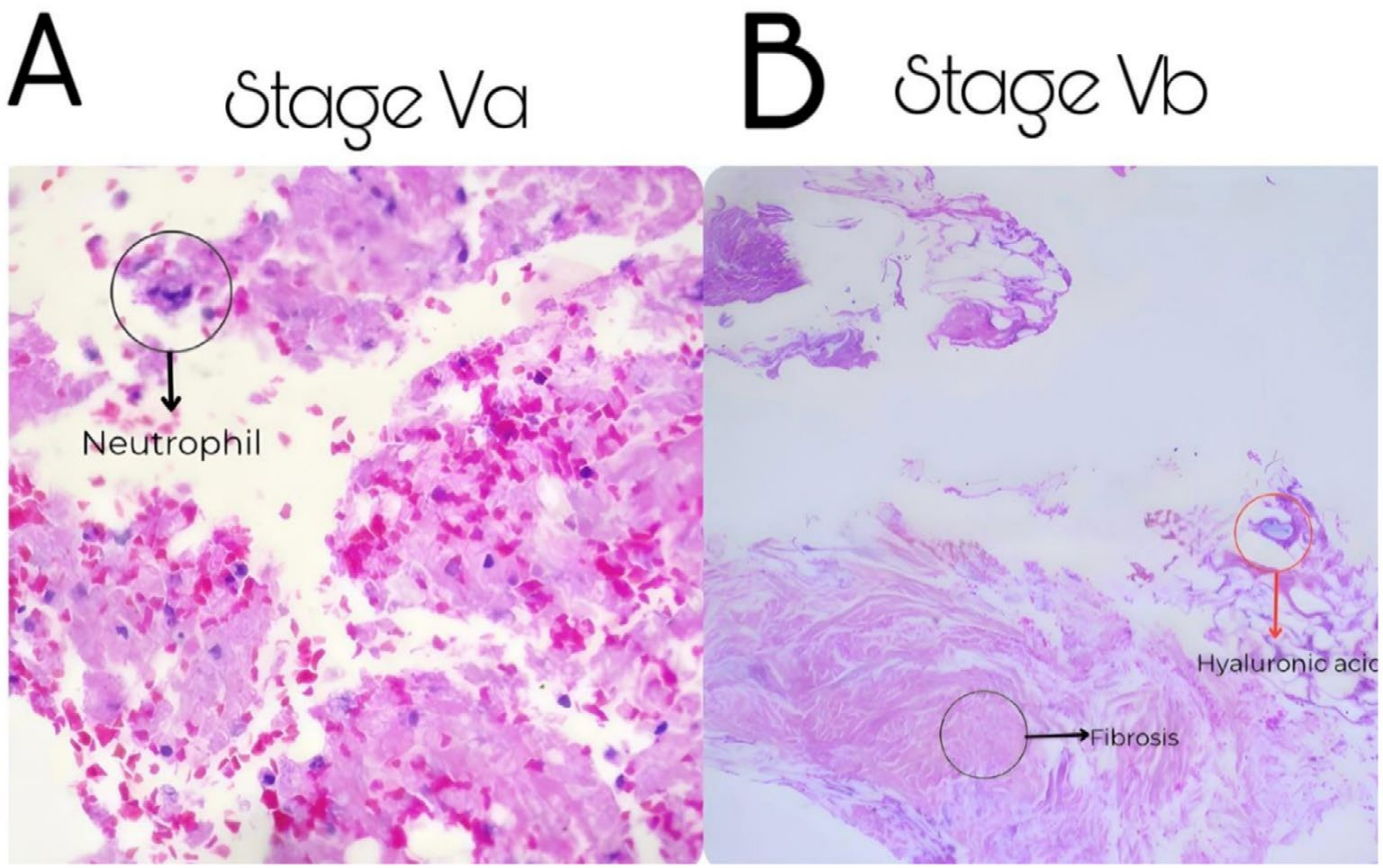
Compound microscopy histopathological findings from necrotic eschar specimens (A) Specimen obtained from a Stage Va necrotic eschar; Dense acute inflammatory infiltrate predominantly composed of neutrophils, fibrin deposition. (B) Specimen obtained from a Stage Vb necrotic eschar; Residual dermal filler material as basophilic amorphous/linear deposits within the dermis, associated with stromal fibrosis.

However, in two clinically severe cases, a tissue sample was obtained to exclude concomitant pathology, including severe infection, viral involvement, or underlying connective tissue disease. The indication for biopsy was based solely on diagnostic clarification in the presence of extensive tissue compromise. In the absence of atypical clinical features or diagnostic uncertainty, histopathological evaluation was not considered mandatory. One specimen obtained from a VO Stage Va necrotic eschar showing extensive coagulative necrosis with dense acute inflammatory infiltrate predominantly composed of neutrophils, fibrin deposition, erythrocyte extravasation, and abundant cellular debris, consistent with active ischemic tissue injury (Figure 3A). A second specimen was obtained from a VO Stage Vb necrotic eschar demonstrating residual dermal filler material as basophilic amorphous/linear deposits within the dermis, associated with stromal fibrosis, collagen bundle thickening, and mild chronic inflammatory infiltrate, consistent with post‐ischemic reparative remodeling and foreign material persistence (Figure 3B). Simple processing and evaluation were performed in the Department of Oral Pathology (institution anonymized for peer review).

### 2.7 Statistical Analysis

Statistical analysis was performed using descriptive statistics. Continuous variables are presented as mean ± standard deviation (SD), and categorical variables as number and percentage. Comparison between ARDIP‐only and ARDIP + adjunctive therapy groups was performed using the non‐parametric Mann–Whitney *U* test due to the small sample size and non‐normal distribution of the data.

## 3 Results

All 20 patients completed the ARDIP and the established follow‐up period. Lesions were classified as VO Stage III (*n* = 4, 20%), Stage IV (*n* = 5, 25%), Stage Va (*n* = 6, 30%), and Stage Vb (*n* = 5, 25%).

**FIGURE 4:**
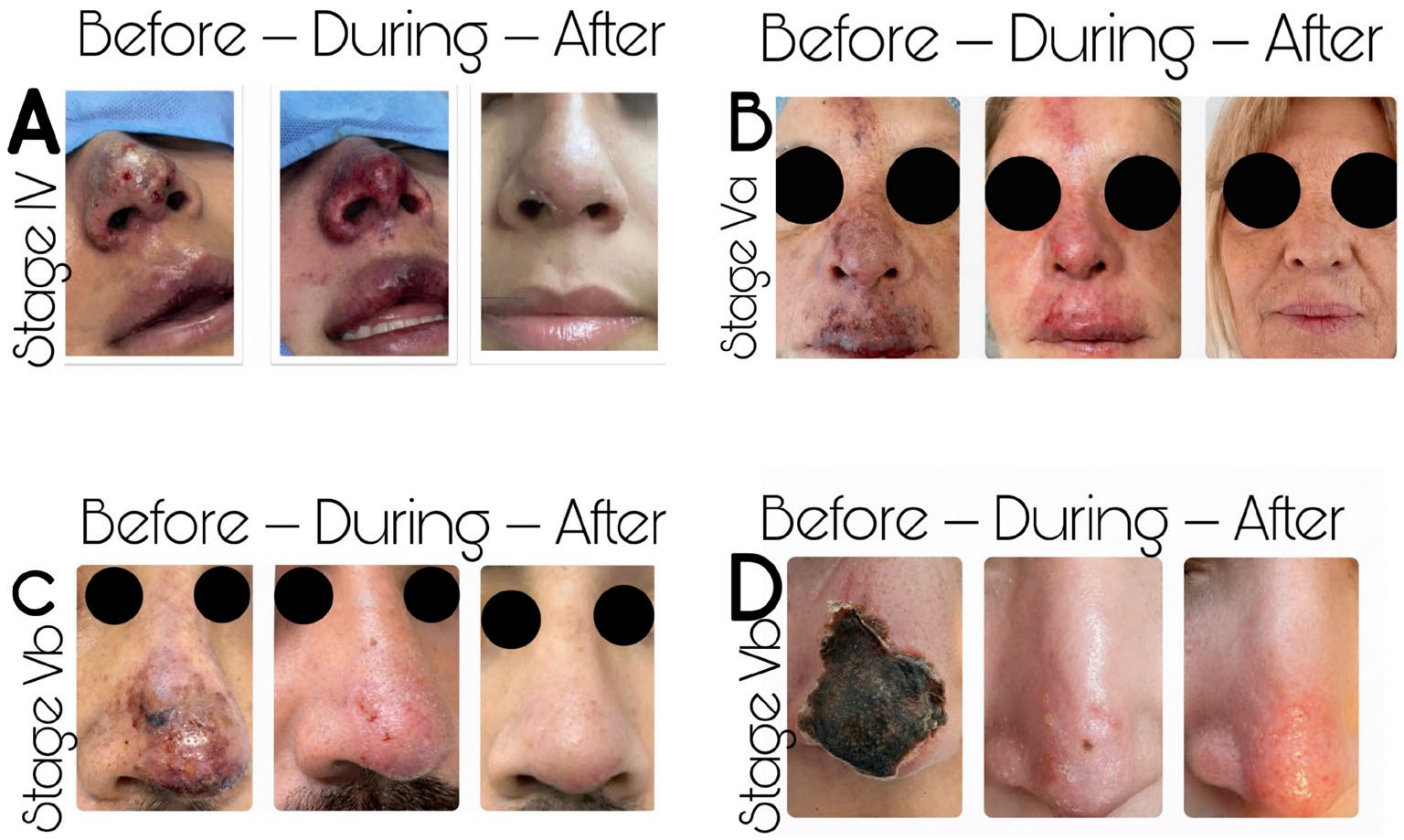
Representative clinical photographs of different VO stage showing signs and skin quality before, during and after treatment with ARDIP. The stages evaluated (A) Stage IV vascular occlusion showing early ischemic skin changes before treatment, intermediate clinical evolution during ARDIP therapy, and complete epithelial recovery after treatment. (B) Stage Va lesion demonstrating progressive tissue reperfusion and resolution following ARDIP intervention. (C) Stage Vb necrotic lesion showing initial ischemic damage, intermediate reparative phase, and final healed outcome. (D) Advanced Stage Vb necrosis with progressive improvement and restoration of skin integrity after treatment.

Complete tissue recovery without visible scarring was achieved in 19 of 20 patients (95%). One patient (5%) presented a mild residual hypopigmented area in the temporal region, without functional impairment or contour deformity. Early‐stage lesions (Stage III‐IV) demonstrated faster clinical recovery, with mean re‐epithelialization occurring within 10–12 days (Figure 4A), whereas advanced necrotic stages (Va‐Vb) required longer recovery periods, averaging 14–18 days (Figure 4B, Figure 4C, Figure 4D). The overall mean treatment duration across the cohort was approximately 15 days. The distribution of GAIS scores among 20 patients included in the study is presented in Table 5. Results are presented as number of patients (n), percentage (%), and mean ± standard deviation.

**TABLE 5:** Distribution GAIS scores after treatment with the ARDIP.

GAIS score	Clinical interpretation	Number of patients (n)	Percentage (%)
1	Exceptional improvement	19	95%
2	Very improved	1	5%
3	Improved	0	0%
4	No change	0	0%
5	Worsened condition	0	0%
Mean ± SD		1.05 ± 0.22	
Total		20	100%

*Note:* Clinical outcomes were assessed using the 5‐point Global Aesthetic Improvement Scale (GAIS), ranging from 1 (exceptional improvement) to 5 (worsened condition). Results are presented as number of patients (n) and percentage (%) of the total cohort (*n* = 20). The mean GAIS score is expressed as mean ± standard deviation (SD), demonstrating the overall aesthetic improvement achieved following completion of the ARDIP.

Thirteen patients were treated exclusively with the ARDIP, while seven received additional regenerative adjuvant therapies.

Mean healing time was 14.2 ± 2.5 days in the ARDIP‐only group and 13.8 ± 2.7 days in the ARDIP + adjuvant therapy group (Table 6).

Statistical comparison between groups was performed using the non‐parametric Mann–Whitney *U* test due to the small sample size and non‐normal distribution of the data.

**TABLE 6:** Clinical characteristics, GAIS outcomes, and healing time according to treatment group.

Variable	ARDIP only (*n* = 13)	ARDIP + adjunctive therapies (*n* = 7)	Total (*n* = 20)
Stage III	3 (23.1%)	1 (14.3%)	4 (20%)
Stage IV	3 (23.1%)	2 (28.6%)	5 (25%)
Stage Va	4 (30.8%)	2 (28.6%)	6 (30%)
Stage Vb	3 (23.1%)	2 (28.6%)	5 (25%)
Score 1–Exceptional improvement	12 (92.3%)	7 (100%)	19 (95%)
Score 2–Very improved	1 (7.7%)	0 (0%)	1 (5%)
Score 3–Improved	0	0	0
Score 4–No change	0	0	0
Score 5–Worsened condition	0	0	0
Mean GAIS score (mean ± SD)	1.08 ± 0.27	1.00 ± 0.00	1.05 ± 0.22
Mean healing time (days, mean ± SD)	14.2 ± 2.5	13.8 ± 2.7	14.1 ± 2.6

*Note:* Distribution of vascular occlusion stages, GAIS outcomes, and mean healing time among patients treated with the ARDIPl, either as a standalone therapy or combined with adjunctive regenerative treatments. Continuous variables are presented as mean ± standard deviation (SD), and categorical variables as number (percentage).

## 4 Discussion

The global expansion of hyaluronic acid dermal filler procedures has been accompanied by a proportional increase in vascular complications. Although uncommon, filler‐induced vascular occlusion represents one of the most severe adverse events in aesthetic medicine and should be considered a true medical emergency. Delayed diagnosis may lead to persistent tissue ischemia, progressive cellular hypoxia, and ultimately irreversible cutaneous necrosis with significant functional and aesthetic consequences. In this context, early diagnosis and immediate intervention are critical to limit tissue damage and preserve vascular integrity [1, 2, 3].

Clinical warning signs such as delayed capillary refill, negative pinprick test, livedoid discoloration, pustule formation, or early eschar development indicate that the ischemic cascade has progressed beyond simple vascular obstruction and entered a self‐propagating biological phase of tissue injury. At this stage, microvascular collapse, endothelial dysfunction, inflammatory amplification, and early thrombotic phenomena contribute to a complex pathophysiological environment that extends beyond simple filler embolization. These mechanisms help explain why delayed or insufficient treatment often results in progressive tissue damage and long‐term sequelae [3].

In the present study, we evaluated the clinical outcomes of the Advanced Regenerative Drainage and Immunomodulatory Protocol (ARDIP), a multimodal therapeutic strategy designed to address both mechanical obstruction and the biological cascade associated with filler‐induced ischemic injury. Our findings demonstrated a high rate of tissue recovery, with complete healing observed in 95% of patients and a mean GAIS score of 1.05 ± 0.22, indicating excellent aesthetic improvement in most cases. Importantly, no systemic or local adverse events were observed during treatment. These results suggest that early implementation of a structured, biologically based protocol may significantly improve outcomes in patients with filler‐induced vascular complications.

Vascular occlusion caused by HA fillers initiates a complex pathophysiological cascade characterized by endothelial dysfunction, microvascular collapse, inflammatory amplification, and oxidative stress. The resulting disruption of tissue perfusion leads to cellular hypoxia and metabolic failure, triggering ischemic injury. Even after restoration of blood flow through Hyal‐mediated reperfusion, secondary damage may occur as part of an ischemia–reperfusion phenomenon characterized by the generation of reactive oxygen species and the release of proinflammatory cytokines. Persistent activation of proinflammatory macrophages amplifies tissue injury, whereas the transition toward the reparative M2 macrophage phenotype promotes angiogenesis, extracellular matrix remodeling, and organized tissue repair. However, excessive or dysregulated repair may activate TGF‐β–mediated signaling pathways, promoting fibroblast overactivation and pathological fibrosis. These mechanisms help explain why inadequately treated ischemic events often result in contractile scarring, contour irregularities, or long‐term aesthetic deformities [7].

Histopathological findings observed in advanced lesions in our study closely reflect these underlying ischemic processes. Examination of biopsy samples demonstrated coagulative necrosis, endothelial disruption, interstitial edema, microvascular thrombosis, and inflammatory infiltrates predominantly composed of proinflammatory macrophages consistent with an M1 phenotype. In later stages, early fibrotic remodeling and thickening of collagen bundles were also identified. Together, these findings correspond to the classic pattern of ischemia‐induced cellular injury followed by dysregulated inflammatory repair. Persistent activation of M1 macrophages sustains the release of proinflammatory mediators such as TNF‐α and IL‐1β, contributing to extracellular matrix degradation and ongoing tissue damage [7, 8, 9].

Current consensus recommendations for the management of HA filler–induced vascular occlusion emphasize the immediate administration of high‐dose hyaluronidase (Hyal) as the primary intervention to restore perfusion and degrade intravascular or extravascular filler material. Expert guidelines from DeLorenzi and the international consensus by Urdiales‐Gálvez et al. highlight the importance of rapid recognition of vascular compromise, repeated Hyal injections, local warming, massage, and adjunctive pharmacologic therapy to reestablish microvascular flow. While these approaches have significantly improved early management of vascular events, they primarily focus on enzymatic reperfusion and do not fully address the complex inflammatory and ischemia–reperfusion cascade that develops in advanced stages of tissue injury [1, 2].

Several clinical algorithms have also incorporated adjunctive systemic therapy, including corticosteroids, antiplatelet agents, vasodilators, and antibiotics when infection is suspected. Although these measures may support tissue recovery, the evidence supporting their use is largely based on expert consensus and case reports. Notably, many existing protocols focus primarily on reversing vascular obstruction, with less emphasis on the subsequent biological processes that contribute to progressive tissue damage, such as microvascular thrombosis, inflammatory amplification, macrophage polarization imbalance, and dysregulated extracellular matrix remodeling [3].

ARDIP was developed to expand upon these established treatment strategies by integrating mechanical decompression, fibrinolytic modulation, and regenerative therapies within a severity‐adjusted multimodal framework. ARDIP addresses both mechanical obstruction and the biological microenvironment of ischemic tissue by combining high‐dose Hyal, controlled drainage techniques, fibrinolytic agents, and regenerative support modalities such as polynucleotides and exosome‐based therapies. This approach aims to restore microvascular perfusion while simultaneously modulating the inflammatory cascade and promoting structured tissue regeneration.

A central component of the ARDIP strategy is active enzymatic and mechanical drainage. Ischemic tissue compartments behave as closed systems characterized by increased interstitial pressure, accumulation of inflammatory exudate, and compromised microcirculation. Mechanical evacuation of necrotic material may reduce inflammatory burden, improve tissue oxygenation, and facilitate restoration of capillary perfusion. This concept parallels the principles described by Gault in the management of extravasation injuries, where decompression and drainage transform a static ischemic environment into a dynamic and recoverable microvascular space [10].

Hyal remains the cornerstone of treatment in HA filler–induced vascular occlusion. Its enzymatic activity degrades HA deposits responsible for intravascular or extravascular compression and promotes interstitial fluid diffusion, thereby reducing tissue tension. The type of hyaluronidase and the physicochemical properties of the injected HA filler may influence enzymatic degradation patterns, as previously described [11]. Adjunctive fibrinolytic agents may further facilitate thrombus dissolution and improve microcirculatory flow by degrading fibrin networks and reducing tissue rigidity. When combined with controlled mechanical drainage, these interventions may enhance tissue reperfusion and reduce inflammatory burden within the ischemic compartment [12, 13, 14]. Corticosteroids may attenuate excessive cytokine release and reduce endothelial activation, while sildenafil improves microvascular perfusion through nitric oxide–mediated vasodilation. Low‐dose aspirin may reduce platelet aggregation and mitigate secondary thrombotic events [15].

Regenerative therapies may further contribute to tissue recovery by modulating cellular repair mechanisms. Polynucleotides (PDRN) exert biological effects through activation of adenosine A2A receptors, promoting fibroblast proliferation, angiogenesis, and extracellular matrix remodeling. Exosomes function as intercellular signaling vesicles capable of delivering microRNAs, cytokines, and growth factors that regulate macrophage polarization and tissue regeneration. Experimental and clinical evidence suggests that these vesicles promote transition toward the reparative M2 phenotype, enhancing granulation tissue formation, accelerating re‐epithelialization, and reducing fibrosis [16, 17, 18, 19, 20]. Well‐established regenerative approaches such as platelet‐rich plasma (PRP) and epidermal growth factor (EGF) have also demonstrated efficacy in promoting angiogenesis and tissue repair [21, 22]. In the present protocol, exosome‐based therapies were incorporated as complementary tools due to their paracrine signaling capacity and immunomodulatory effects. However, these modalities should be interpreted within a broader regenerative framework that includes PRP and EGF as validated therapeutic alternatives.

Additional supportive therapies such as photobiomodulation, ozone therapy, and hyperbaric oxygen therapy may further enhance tissue recovery by optimizing oxygen delivery, stimulating mitochondrial activity, and promoting angiogenesis. These modalities have been shown to accelerate wound healing and reduce inflammatory signaling in ischemic tissues. In the context of filler‐induced vascular compromise, these therapies may help restore microvascular function and improve tissue viability during the reparative phase [23, 24, 25, 26, 27].

Despite the encouraging results observed in this study, several limitations must be acknowledged. The study design consisted of a non‐randomized case series with a relatively small sample size, which may limit generalizability. Additionally, adjunctive regenerative therapies were implemented based on clinical severity rather than through a standardized comparative design. Therefore, future controlled studies are warranted to better define the individual contribution of each therapeutic component and to validate the ARDIP in larger patient populations.

Overall, these findings suggest that early implementation of a biologically based multimodal therapeutic strategy may improve tissue survival, accelerate healing, and reduce long‐term complications associated with HA filler–induced vascular occlusion.

## 5 Conclusions

Filler‐induced VO leading to tissue ischemia and necrosis should be considered a true medical emergency in aesthetic practice. Rapid recognition and early intervention are essential to prevent irreversible tissue damage.

The ARDIP addresses the key pathophysiological mechanisms involved in sustained tissue hypoxia by combining enzymatic reperfusion, mechanical decompression, fibrinolysis, and regenerative immunomodulation within a severity‐adjusted treatment framework.

Early implementation of a biologically informed multimodal protocol such as ARDIP may represent a promising therapeutic strategy for improving tissue survival, accelerating wound healing, and reducing long‐term fibrotic sequelae following filler‐induced vascular complications. Importantly, manual drainage in this context should be interpreted as a controlled decompressive maneuver rather than forceful compression, minimizing the risk of further vascular compromise in fragile ischemic tissues.

Future prospective controlled studies are required to further validate the efficacy of multimodal regenerative protocols in the management of filler‐induced vascular complications.

## Author Contributions

Macarena Olivares conceived and designed the study. Macarena Olivares and Diego Araya performed the clinical procedures and patient management. Eloisa Forero and María José Benoit contributed to data collection and organization. Victor Mercado contributed to data interpretation and critical revision of the manuscript. Macarena Olivares drafted the manuscript. All authors reviewed, edited, and approved the final version of the manuscript.

## Funding

This research did not receive any specific grant from funding agencies in the public, commercial, or not‐for‐profit sectors. All procedures and materials were funded by the authors.

## Ethics Statement

This study was reviewed and approved by the Ethics Committee of Faculdade do Oeste Paulista (FACOP), Brazil (IRB approval No. 0016–2026). All procedures were performed in accordance with the Declaration of Helsinki and its subsequent amendments.

## Consent

Written informed consent was obtained from all patients included in this study for participation and publication of their clinical data. The patients were fully informed about the nature of the procedures, potential risks, benefits, and alternatives. All procedures were conducted in accordance with the ethical standards of the institutional and/or national research committee and with the Declaration of Helsinki. Written informed consent was obtained from the patients for the publication of clinical images included in this article. Patients understood that their images may be published in a scientific journal and made publicly available. All reasonable efforts have been made to protect patient identity, and identifying information has been removed or anonymized.

## Conflicts of Interest

The authors declare no conflicts of interest.

## Data Availability

The data that support the findings of this study are available on request from the corresponding author. The data are not publicly available due to privacy or ethical restrictions.
